# Rat sensorimotor cortex tolerance to parallel transections induced by synchrotron-generated X-ray microbeams

**DOI:** 10.1038/s41598-017-14757-3

**Published:** 2017-10-30

**Authors:** Erminia Fardone, Alberto Bravin, Alfredo Conti, Elke Bräuer-Krisch, Herwig Requardt, Domenico Bucci, Geraldine Le Duc, Giuseppe Battaglia, Pantaleo Romanelli

**Affiliations:** 1 European Synchrotron Radiation Facility, Grenoble, France; 2 Department of Neurosurgery, University of Messina, Messina, Italy; 3 I.R.C.C.S. Neuromed, Pozzilli, Italy; 4 Centro Diagnostico Italiano, Brain Radiosurgery, Cyberknife Center, Milano, Italy; 5 AB Medica, Lainate, Italy; 6 Present Address: Department of Biological Science and Program in Neuroscience, Florida State University, Tallahassee, FL USA

## Abstract

Microbeam radiation therapy is a novel preclinical technique, which uses synchrotron-generated X-rays for the treatment of brain tumours and drug-resistant epilepsies. In order to safely translate this approach to humans, a more in-depth knowledge of the long-term radiobiology of microbeams in healthy tissues is required. We report here the result of the characterization of the rat sensorimotor cortex tolerance to microradiosurgical parallel transections. Healthy adult male Wistar rats underwent irradiation with arrays of parallel microbeams. Beam thickness, spacing and incident dose were 100 or 600 µm, 400 or 1200 µm and 360 or 150 Gy, respectively. Motor performance was carried over a 3-month period. Three months after irradiation rats were sacrificed to evaluate the effects of irradiation on brain tissues by histology and immunohistochemistry. Microbeam irradiation of sensorimotor cortex did not affect weight gain and motor performance. No gross signs of paralysis or paresis were also observed. The cortical architecture was not altered, despite the presence of cell death along the irradiation path. Reactive gliosis was evident in the microbeam path of rats irradiated with 150 Gy, whereas no increase was observed in rats irradiated with 360 Gy.

## Introduction

Microbeam radiation therapy (MRT) is a developing technique using X-ray microplanar beams (also known as microbeams) generated by a synchrotron radiation X-ray source, with potential translation to humans. The pioneering steps of this technique were carried out at the Brookhaven National Laboratory (Upton, NY, USA) and then developed and refined at the European Synchrotron Radiation Facility (ESRF, Grenoble, France)^1^. It has been demonstrated that doses up to 600 Gy and 200/300 Gy are well tolerated by normal brain tissue if delivered respectively by thin (25 to 100 μm) and thick (up to 600 μm) microbeams^2–4^. It has been shown that the overall structure of normal tissues between the paths of the microbeams remains intact^3^, thus preserving neurological functions^5^. The experimental research in the field of MRT has regarded the treatment of aggressive tumours^6–11^. Nonetheless, recent works have shown that X-ray microbeams can be used also for neuromodulation^12^ in particular to treat drug-induced^13^ or genetically-generated epilepsy^14^ in experimental animal models. The pathophysiological bases of this treatment is the use of microbeams to produce cerebral cortex transections in order to segregate cortical columns by sawing their horizontal connections, similarly to what is produced by the surgical non-resective technique named multiple subpial transections (MSTs). MSTs are employed to treat selected cases of focal medically refractory epilepsy involving eloquent cortices^15,16^. Such microbeam transections provide a highly innovative and non-invasive way to generate cortical microscopic cuts of specific size and spacing, offering a novel attractive tool to study cortical function and to develop new approaches for the treatment of drug-resistant focal epilepsy.

Growing evidence show the remarkable tolerance of the brain to thin microbeams (25 to 100 μm) delivering doses up to 1000 Gy. However, thick beams, also called minibeams^17,18^ are much more likely to find clinical application because they could be more easily produced by sources different from synchrotrons^19^, thus markedly facilitating the technique application by medium-size hospitals. To this aim, here we characterized the radioresistance of brain cortex to parallel transections induced with thin (100 µm) or thick (600 µm) beams, delivering 360 or 150 Gy, respectively, in healthy rats.

## Results

### Behavioural analysis

**Figure 1:**
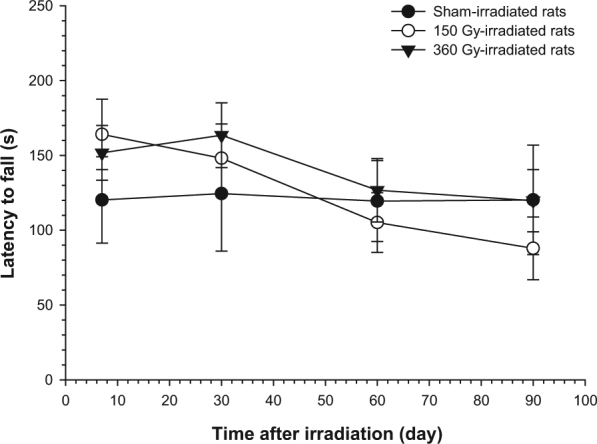
Rotarod test in sham and irradiated rats over a 3-month observation period. Values were means ± S.E.M. of 4–8 rats for each group.

Irradiation of the sensorimotor cortex did not induce any gross sign of paralysis or paresis over a 3-month period. Motor performance, assessed 7 days after irradiation and then every month for 3 months by the rotarod test, showed a small reduction of motor impairment in the group of 150 Gy irradiated rats, which, however, did not reach statistical significance (Fig. 1). Body weight curves did not show any alteration of weight gain (data not shown).

### Histological analysis

**Figure 2:**
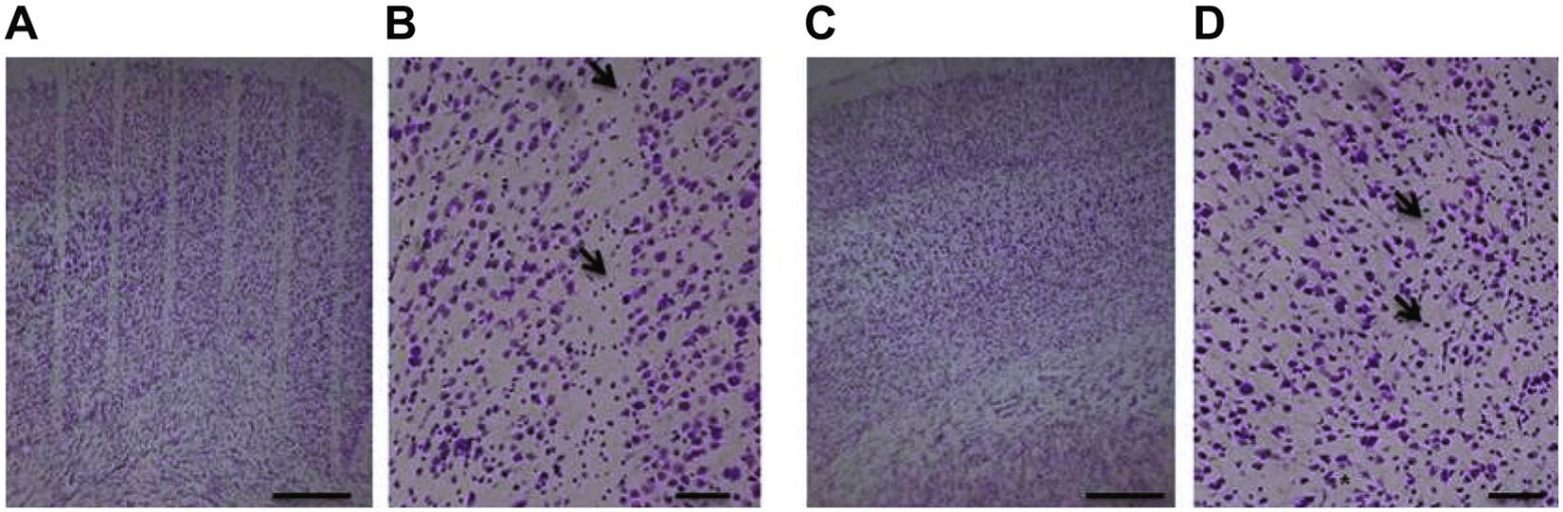
Nissl staining of cerebral cortex of rats at 3 months after irradiation. Parallel cortical transections (generated by an array of microbeams, 100 µm wide and spaced by 400 µm, incident peak dose: 360 Gy) are clearly visible (**A**,**B**). The beam paths are less visible in the cerebral cortex of rats irradiated with an array of 4 minibeams (600 µm wide, 1200 µm spacing, incident peak dose: 150 Gy) which do not generated clear-cut transections (**C**,**D**). Black arrows indicate cells along the path. Scale bars: 500 µm in A and C; 100 µm in B and D.

Nissl staining of the cerebral cortex showed that the cytoarchitecture of the cortex was entirely preserved in 360 and 150 Gy irradiated rats. We could detect almost complete cell loss only along the microbeam paths of 360 Gy irradiated rats, whereas in brains of 150 Gy irradiated rats there was a partial loss of cells (Fig. 2A–D). The cortical volume irradiated by a microbeam path is visible as a microsurgical incision along the entire brain, with sharp margins, whereas the adjacent cortex shows no sign of damage. There is no sign of cellular damage and/or radionecrosis in the “valley” cortex.

**Figure 3:**
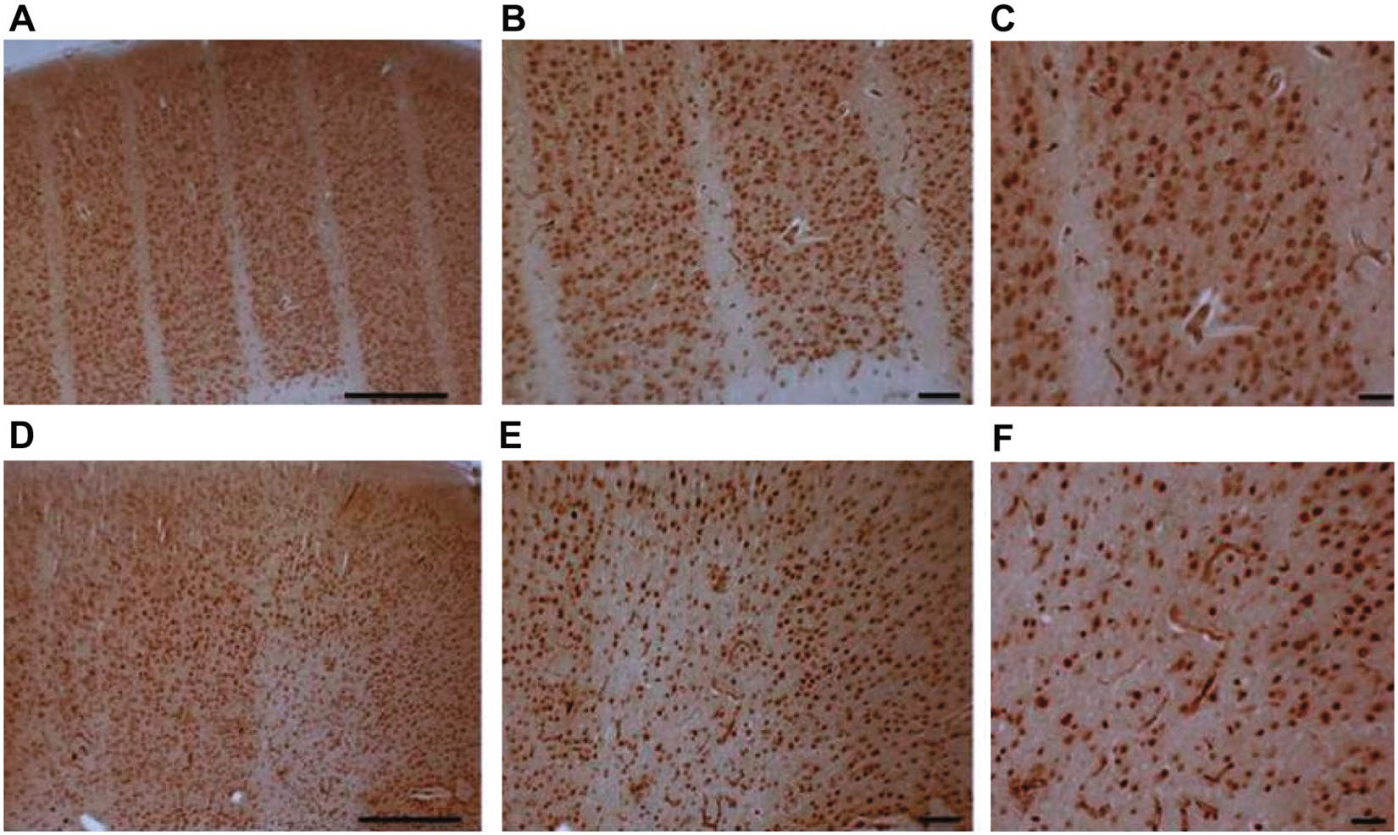
Immunohistochemistry of NeuN in the rat brain cortex 3 months after irradiation. Brain cortex irradiated with 360 Gy (**A**–**C**) or 150 Gy (**D**–**F**). Scale bars: 500 µm in A and D, 200 µm in B and E, 100 µm in C and F.

Immunohistochemistry of the neuronal marker NeuN^20^ confirmed the loss of neurons within the irradiation paths of rats irradiated with 360 Gy, whereas neurons outside the irradiation path were entirely preserved (Fig. 3A–C). Rat brains irradiated with 150 Gy show a partial loss of neurons along the irradiation path (Fig. 3D–F).

**Figure 4:**
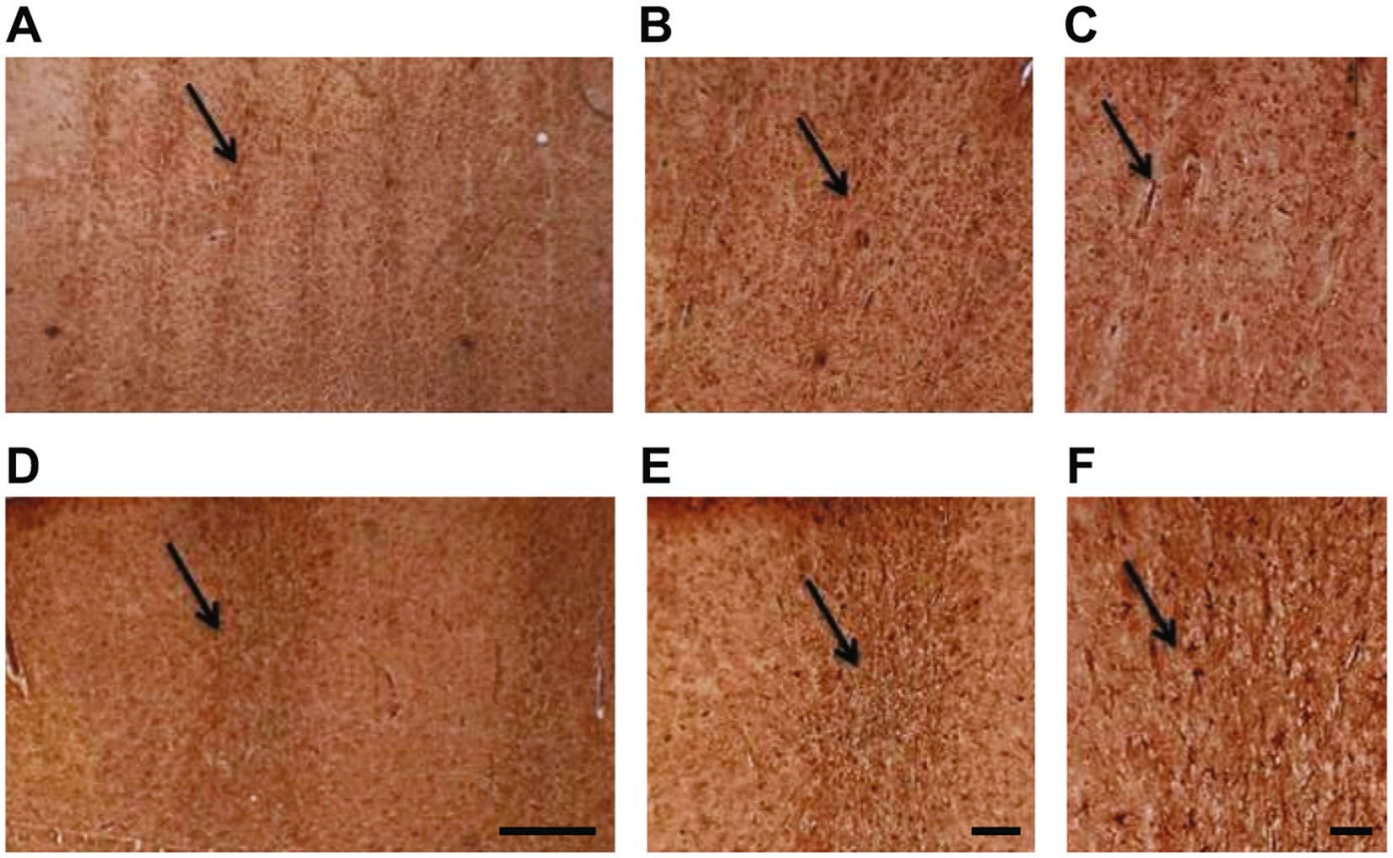
Immunohistochemistry of GFAP in cortical sensorimotor cortex of rats 3 months after irradiation. Brains of 360 Gy irradiated rats (**A**–**C**) and 150 Gy irradiated rats (**D**–**E**). Black arrows identify the microbeam path. Scale bars: 500 µm in A, 200 µm in B and 100 µm in C and F.

To examine the reaction of astrocytes to damage induced by irradiation, we performed immunohistochemistry for the glial fibrillary acidic protein (GFAP), a marker of astrocytes^21^. Three months after irradiation, a light GFAP immunoreactivity was observed within the beam paths, but not outside, in rats irradiated with 360 Gy (Fig. 4A–C). An intense GFAP immunoreactivity was observed within the beam paths in rats irradiated with 150 Gy (Fig. 4D–F) suggesting that irradiated tissues induced a reactive gliosis depending on the deposited dose. In our experimental conditions, we show that large dose of radiation deposited in a narrow region induces a light reactive gliosis, whereas a lower dose, delivered to a much larger brain region, induced an intense activation of quiescent astrocytes within the irradiated area, with preservation of tissue integrity and absence of radionecrosis.

## Discussion

Our data show that microbeam radiosurgery generates histologically-visible sensorimotor cortex microtransections and that these transections are compatible with the preservation of the motor function. The ability to generate, with a non-invasive approach, histologically neat transections of microscopic size over motor cortex, without inducing alterations of motor function, is a remarkable achievement. Actually, the obtained transections mimic the surgical MST technique, which aims at cutting the horizontal connections mediating epileptic activity diffusion from the ictal focus to adjacent and distant cortices^22–25^. The cerebral cortex is actually functionally organized in vertically oriented columns of neurons working as a homogeneous processing unit. The output of the columnar networks is mainly transmitted by vertical axons directed to near or far cortical regions, to the thalamus and basal ganglia, or to the brainstem and spinal cord; adjacent columns are instead interconnected by horizontal axons, providing the recruitment and synchronization of the critical mass of cortex needed to generate seizures^26–29^. The spread of epileptic activity follows a non-uniform horizontal spatial pattern involving the cortical layer V, which acts as the seizure trigger^30,31^. MST provides a way to undercut the horizontal axons, mediating the spread of epileptogenic activity while sparing the vertically-oriented fibers subserving neurological function. With surgical MST, cortical transections are spaced approximately 5 mm apart and oriented perpendicular to the long axis of the selected epileptic gyrus. Multiple parallel transections made at this distance over a cortical gyrus provide an effective disconnection and parcellization of the seizure focus without injury to, or disruption of, the basic functions of the columns^15^. Synchrotron-generated microtransections offer a new, low-invasive way to perform MST. The synchrotron radiation beams are well suited to treat superficial targets such us the cortex. This approach could be even applied to non-eloquent cortex and to the hippocampus allowing these structures to retain residual function in the brain region where the seizure focus is located. It has been already shown that microbeam transections stop seizures originating from eloquent cortex in an experimental model of epilepsy^13,32^. Microbeam transections, either placed over neocortical seizure foci could be an excellent tool to be added to the current radiosurgical techniques used to control seizures. Here we provide also an immunohistological characterization of microtransections performed on the primary motor cortex. Current stereotactic radiosurgical devices such as Gamma Knife^®^, Cyberknife^®^ or Linacs^®^^33–35^ cannot provide a beam size smaller than 4 mm and therefore cannot generate the equivalent of a surgical incision on a microscopic scale. Synchrotron radiation offers also a much steeper dose fall out and the ability to deliver doses much higher to much smaller volumes than any other radiation technique^13^. Here we have tested 2 different beam sizes: 100 and 600 µm delivered to healthy adult rats. Histologically neat microtransections have been generated within the sensorimotor cortex without signs of motor damage up to 3 months after MRT. The reactive gliosis was observed in rats irradiated with beams of 600 μm and a dose of 150 Gy suggesting an astrocyte reaction to irradiation. Post-radiation gliosis is a known phenomenon. Indeed, it has been demonstrated, after whole brain irradiation with 15 Gy, the induction of reactive gliosis since the early days after exposure^36^. Reactive gliosis after microbeam irradiation has also been showed 30 days after microplanar irradiation with 200 Gy^12^. Here, we show that a high dose (360 Gy) deposited by microbeams in a narrow region induced a minimal reactive gliosis, whereas a low dose (160 Gy) delivered by much broader beams induced an intense reactive gliosis within the irradiated area still visible 3 months after irradiation. Overactivation of the glial system is commonly detrimental to neurons as it includes induction of quiescent astrocytes into reactive astrocytes that can aggregates at any site of insult to produce gliosis. Microbeam irradiation (360 Gy) induced cell death along the path, but we did not observe reactive gliosis at 3 months, suggesting a “clean” removal of dead cell by activated astrocytes along the irradiation path and preservation of inter-beam neurons, as activated astrocytes go back to their quiescent state. This is an important aspect as reactive gliosis scars deriving from any insult or damage to the nerve tissue is harmful to neuronal function and can alter neuronal circuitry. The reason to test a larger beam size (600 μm) relies on the fact that new emerging technologies could allow a clinical transfer of submillimetric beams without the need of a synchrotron. According to our results, thick beams (600 μm) provide satisfactory results in terms of absent radiation-induced detrimental effects in the tissue adjacent the cortical transections, specifically in terms of function preservation, whereas gliosis between the sections may hypothetically protect against regeneration of horizontal axons. The 80-to-20% dose fall-off predicted for synchrotron X-ray of 120-keV median energy is 30-μm. This value would actually be increased to 0.2–0.5 mm with beams of the same size but produced by orthovoltage sources, still having a favourable profile for a multiple subpial transection. Even though we recognize that further studies are required, we consider this as an important result, since submillimetric beam radiotherapy will be conceivably delivered by high energy orthovoltage X-ray sources in the future^37,38^ and, therefore, used in the clinical setting.

## Methods

### Ethical statement

All procedures related to animal care conformed to the Guidelines of the French Government (licenses 380325 and B3818510002) and were performed in accordance with French laws and the 2010/63/UE directive for animal experimentation and were approved by the ESRF Internal Evaluation Committee for Animal Welfare and Rights.

### Animal preparation and housing

Adult male Wistar rats (250–270 g, Charles River Laboratories, L’Arbresle, France) were used. Rats were maintained under controlled environmental conditions (temperature of 22 ± 2 °C, 40–60% humidity and a 12-hour light/dark cycle) with food and water *ad libitum*. All efforts were made to minimize the potential sufferance and discomfort of animals and their number. Rats were anesthetized in with 5% isoflurane for induction and followed by an intraperitoneal injection of xylazine/ketamine (64.5/5.4 mg*kg^−1^) for maintenance.

### X-ray beams generation

Experiments were performed at the ID17 Biomedical Beamline of the European Synchrotron Radiation Facility (ESRF). X-rays are emitted by the wiggler source located in the straight section of the storage ring. The wiggler produces a continuous (white) beam spectrum filtered for this study by a succession of five attenuators (Be (0.5 mm), C (1.5 mm), Al (3.0 mm), Cu (1.0 mm) resulting in a photon spectrum which extends from about 50 to 350 keV (mean energy of 105 keV)^39^. The quasi-laminar beam was spatially fractionated into an array of microbeams of variable size by using an adjustable multislit collimator^40^.

### Irradiation and group allocation

**Figure 5:**
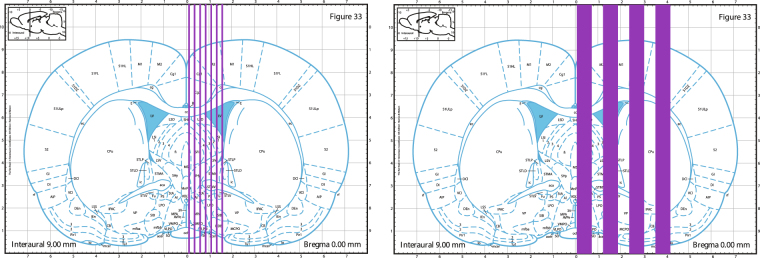
Brain regions of rats irradiated with an array of 7 microbeam, 100 µm wide, and spaced by 400 µm with an incident peak dose of 360 Gy (left image) or an array of 4 minibeams, 600 µm wide and spaced by 1200 µm with an incident peak dose of 150 Gy (right image). Blue lines indicate the beam array. The irradiation extension in antero-posterior direction was 4 mm (1 mm anterior to −3 mm posterior to the bregma); in the medio-lateral direction, the microbeam array extension was respectively 1.5 and 4.2 mm for 360 Gy and 150 Gy, respectively. Images are taken from Paxinos and Watson rat atlas^45^.

Rats were placed on a custom-made stereotactic frame fixed on a Kappa goniometer, by which the rat could be translated and rotated in front to the fixed horizontal X-ray beam. The spatial configuration of the microbeams was checked by gafchromic^®^ films. Irradiations covered 4 mm on the rostrocaudal direction, from 1 mm anterior to 3 mm posterior to the bregma, in both irradiated groups (Fig. 5). Mediolateral irradiation covered a 1.5 mm span for the group receiving 360 Gy and a 4.2 mm span for group receiving 150 Gy.

Rats were randomly assigned to three different groups: 8 rats were irradiated with 7 microbeams, 100 μm wide, 400 μm centre-to-centre (c-t-c), with a skin entrance peak dose of 360 Gy and a valley dose of 5.3; 8 rats were irradiated with 4 “thick” microbeams, 600 μm wide and 1200 μm c-t-c with a skin entrance peak dose of 150 Gy and a valley dose of 6 Gy. An additional group of 8 rats was sham-irradiated and used as control group.

### Dose calculation and Monte Carlo simulation

The dose distribution was calculated using the Monte Carlo code PENELOPE 2006^41^. PENELOPE simulates the coupled transport of photons, electrons and positrons in the energy interval from 50 eV to 1 GeV, and in arbitrary material systems. PENELOPE has been widely used in the medical physics field^42^ and in MRT dose assessment^17,39,43^. It uses a mixed simulation scheme in which hard interactions are simulated collision by collision and small angular deflections and energy losses are treated in a grouped manner. Since the working energy range is a few hundreds of keV, the most relevant interactions are photoelectric effect and Compton scattering. In this work the number of primary photon stories was 2*10^8^ in all the calculations.

The simulations have been performed using a rat head phantom. It was modelled by three concentric ellipsoids whose volumes were 13.00 cm^3^ for the brain, 3.13 cm^3^ for the skull, and 7.15 cm^3^ for the skin; these values have been extracted from MRI images acquired on the rats of the same strain and weight; the skin thickness was 0.7 mm and the cranial bone thickness of 0.4 mm^43^. The parameter that characterized the dose deposition is called Peak-to-Valley Dose Ratio, namely the ratio between the dose value at the centre of the peak and the dose value in the region in between two adjacent peaks, also indicated as valley dose.

### Behavioural analysis

Control and irradiated rats were placed in large transparent Plexiglas boxes hosting two animals each and observed daily for the week following the irradiation and then weekly. The evolution of their body weight was noted each month and neurological observations (in particular signs of contralateral hemiparesis) were performed once a week.

Motor behaviour was assessed by the rotarod (PanLb/Harvard, les Ulis, France) in order to evaluate motor coordination after sensorimotor cortex irradiation. The rotarod test assesses fine motor coordination by requiring an animal to maintain balance on an accelerating rotating rod^44^. Rats were first habituated to low speed for 30 s. Seven, 30, 60 and 90 days after irradiation, sham-irradiated and irradiated rats underwent to 3 trials/day of 4 to 40 RPM at constant acceleration for 5 min, with a 10-min resting interval between them. The latency to fall was recorded.

### Histology and immunohistochemistry

Rats were sacrificed 3 months after irradiation with an overdose of Dolethal^®^; brains were rapidly dissected out and fixed in Carnoi (ethanol: acid acetic: chloroform; 6:1:3). Twenty-four hours later, brains were placed in 70% ethanol until they were included in paraffin. Fifteen µm coronal brain sections were cut using a microtome (RM2245 semiautomatic Leica, Milan, Italy) and stained with 0.1% thionin. For immunohistochemistry, sections were incubated overnight with an anti-NeuN monoclonal mouse antibody (1:1000; Chemicon, Billerica, MA, USA) and an anti-GFAP monoclonal mouse antibody (1:100; Sigma-Aldrich, Saint-Quentin Fallavier, France) and then, for 1 h, with secondary biotin-coupled anti-mouse antibodies (1:200; Vector Laboratories, Burlingame, CA, USA). Control staining was performed without the primary antibodies. Sections were examined by bright field microscopy and images were captured with the Olympus BX51 optical microscope, equipped for epifluorescence and objectives 10X, 20X and 40X, connected to a video camera. Images were processed by the software Cell^B (Olympus microscope, Life science instrument, France).

### Statistical analysis

All statistical analyses were performed using the GraphPad Prism software program (version 5.0, GraphPad Software, San Diego, CA, USA). Values are means ± S.E.M. Level of significance was set at p < 0.05. Two-Way ANOVA was used for analysis in Fig. 1.

## Conclusions

Data presented here show that irradiations with synchrotron-generated microbeams were well tolerated up to 3 months and did not induce motor deficits in healthy rats, despite the cell loss present in the beam paths. Despite further studies are necessary, microradiosurgical transections may represent a novel tool for the treatment of brain disorders such as drug-resistant epilepsy with epileptogenic focus laying in a critical brain region. Technologies are emerging that could allow a clinical use of thick beams without the need of a synchrotron allowing the adoption of a similar strategy in a hospital setting.
